# Investigation of a Minocycline-Loaded Nanoemulgel for the Treatment of Acne Rosacea

**DOI:** 10.3390/pharmaceutics14112322

**Published:** 2022-10-28

**Authors:** Ayesha Siddiqui, Pooja Jain, Thompson Santosh Alex, Mohammed Asgar Ali, Nazia Hassan, Jamshed Haneef, Punnoth Poonkuzhi Naseef, Mohamed Saheer Kuruniyan, Mohd. Aamir Mirza, Zeenat Iqbal

**Affiliations:** 1 Department of Pharmaceutics, School of Pharmaceutical Education and Research, Jamia Hamdard, New Delhi 110062, India; 2 Department of Pharmaceutical Chemistry, School of Pharmaceutical Education and Research, Jamia Hamdard, New Delhi 110062, India; 3 Department of Pharmaceutics, Moulana College of Pharmacy, Perinthalmanna 679321, India; 4 Department of Dental Technology, College of Applied Medical Sciences, King Khalid University, Abha 61421, Saudi Arabia

**Keywords:** o/w nanoemulsion, minocycline, eucalyptus oil, Carbopol 940, permeation study

## Abstract

In the present investigation, a nanoemulgel of minocycline was formulated and optimized for an improved drug delivery and longer retention time in the targeted area. Combining eucalyptus oil, Tween 20, and Transcutol HP, different o/w nanoemulsions were formulated by the oil phase titration method and optimized by pseudo-ternary phase diagrams. The morphology, droplet size, viscosity, and refractive index of the thermodynamically stable nanoemulsion were determined. Furthermore, optimized nanoemulsion was suspended in 1.0% *w*/*v* of Carbopol 940 gel to formulate the nanoemulgel, and for this, pH, viscosity, and spreadability were determined and texture analysis was performed. To compare the extent of drug penetration between nanoemulsion and nanoemulgel, ex vivo skin permeation studies were conducted with Franz diffusion cell using rat skin as the permeation membrane, and the nanoemulgel exhibited sustained-release behavior. It can be concluded that the suggested minocycline-containing naoemulgel is expected to treat acne rosacea more effectively.

## 1. Introduction

Acne is a widely prevalent disorder of pilosebaceous unit-hair follicles in the skin [1]. Secretion of sebum by the oil glands is a natural skin phenomenon that helps to keep the skin moist [2]. Clogging of the oil glands may eventually lead to pimples and cysts.

Patient with mild acne is diagnosed by the presence of minor pimples, whiteheads, blackheads, and comedones. Moderate acne is characterized by severe papules/pustules, comedones, and cystic nodules that usually leave behind scars on the skin [3]. The distinguished symptoms of rosacea from acne are either transient or persistent facial erythema, visible blood vessels [4], papules, and pustules [5]. Based on these physical patterns, rosacea is classified into four subtypes: erythematotelangiectatic, papulopustular, phymatous, and ocular [6].

The disease etiology of rosacea is still under many wraps, which paves the way for diverse research avenues. Several hypotheses document the potential role of vascular abnormalities, dermal matrix degeneration, environmental factors, and microorganisms, such as *Demodex folliculorum* and *Helicobacter pylori* [7], in the condition of rosacea.

The topical modalities against acne rosacea are directed to four major areas involved in disease progression: keratinous plugs in sebaceous ducts, large sebaceous glands producing excess sebum, increased numbers of resident follicular bacteria, and inflammatory response to chemical mediators passing through the follicular wall. The conventional antibiotics used are erythromycin, clindamycin, metronidazole, and sodium sulfacetamide. Skin permeation enhancers, such as salicylic acid (0.5 to 2%) in the form of a gel, lotion, cream, or solution, are also used [8]. Some of the marketed topical preparations are Finacea gel, Metrogel, Avar LS cream, brimonidine topical, and so on [9]. For systemic delivery, antibiotic agents, such as tetracycline, erythromycin, ampicillin, metronidazole, and minocycline, are much commonly prescribed [10]. In the aspect of lifestyle, practicing sun avoidance behaviors and the use of broad-spectrum sunscreens have central importance to rosacea management [11]. 

In order to eliminate the first-pass metabolism of minocycline, there is a need for a suitable drug delivery system through which the desired effect is achieved without compromising its efficacy. Since topical delivery has an edge over the systemic route in terms of elimination of first-pass metabolism, locoregional application, and intimate contact with the affected site, various researchers have carried out numerous studies to administer minocycline topically [12] for the treatment of facial papulopustular rosacea [13]. Minocycline nanoemulgel is one such directed approaches conceived for the present work. An o/w nanoemulsion of minocycline was formulated with eucalyptus oil as an internal phase and dispersed in a Carbopol 940 gel base. Eucalyptus oil may cause high irritation to an already-compromised skin if taken as an external phase [14]. The purported formulation may expectably improve dermal drug absorption, aid the delivery of poor lipophilic drug, enhance drug retention time [15] in the targeted area, and show fewer side effects as compared with systemic delivery [14,16]. 

## 2. Materials and Methods

### 2.1. Materials

Minocycline was procured from Sun Pharmaceuticals Industries Ltd., Gurugram, India; Carbopol 940 was from Lubrizol Advanced Materials India Pvt. Ltd. (Mumbai, India); and Cremophor EL was purchased from Sigma-Aldrich (St. Louis, MO, USA). Tween 80, Tween 40, Tween 20, and propylene glycol were taken from Merck Pharmaceuticals, Mumbai, India. Transcutol HP were the gift samples obtained from Gattefosse India Pvt. Ltd. Mumbai, India ethanol and propan-2-ol were procured from SD Fine Chem Ltd., India, Mumbai, Maharashtra. Olive oil, rose oil, castor oil, and eucalyptus oil were of appropriate purity grades, as needed and purchased from local vendors.

### 2.2. Screening of Oils

The solubility of minocycline in various oils was determined by adding an excess amount of drug in 2 mL of individual oil (olive oil, rose oil, castor oil, eucalyptus oil) in 5 mL capacity stopper vials, ref. [17] and mixed using a vortex mixer. The mixture vials were then kept in a biological shaker at 25 ± 1.0 °C for 72 h. The samples were removed and centrifuged at 3000 ± 50 rpm for 15 min. The supernatant obtained was passed through a 0.22 μm nylon syringe filter, dissolved in methanol [18], and analyzed using a UV-VIS spectrophotometer (UV 1601, Shimadzu, Japan) at 254 nm.

### 2.3. Screening of Surfactant and Cosurfactant

The screening of surfactants (Cremophor EL, Tween 40, Tween 80, Tween 20) and cosurfactants (propylene glycol, ethanol, propan-2-ol, Transcutol HP) was carried out on the basis of saturation solubility of minocycline in respective surfactants and cosurfactants. In a 5 mL stoppered vial, an excess amount of drug was added to 1 mL of selected surfactant and cosurfactant and was kept it in a mechanical shaker at 25.0 ± 0.5 °C for 72 h to reach equilibrium. The contents were then centrifuged using a high-speed centrifuge (Remi Instruments, Delhi, India) at 3000 rpm ± 50 rpm for 30 min at 37 ± 0.5 °C. The collected supernatant was dissolved in methanol and analyzed using a UV spectrophotometer (Shimadzu, Kyoto, Japan) at 254 nm for which the linearity range was found to be between 2 and 18 µg/mL, and LOQ was 1 µg/mL. The solubility studies were conducted in a triplicate manner, and results were reported as mean ± SD.

### 2.4. Pseudo-Ternary Phase Diagram Construction

Pseudo-ternary phase diagrams were constructed with the aqueous titration method to determine the concentration range of components for the existing boundary of nanoemulsions (NEs). Five phase diagrams were prepared with 1:1, 1:2, 2:1, 4:1, and 5:1 weight ratios of Tween 20/Transcutol HP [19]. The surfactant and cosurfactant used (i.e., Tween 20 and Transcutol HP) were sufficient to attain the required HLB to emulsify the oil (required HLB of oil = 9.8) within these ratios. The aqueous phase and the surfactant mixture were then mixed at weight ratios of 1:9, 1:8, 1:7, 1:6, and 1:5 (*w*/*w*). These weight ratios of oil and S/CoS were diluted dropwise with water under moderate agitation. After being equilibrated, the mixtures were assessed visually [20].

### 2.5. Method of Formation of Nanoemulsion

Minocycline-loaded nanoemulsion was a formulation by the aqueous titration method. For this, selected formulation components based on a pseudo-ternary phase diagram were vortexed for few minutes to obtain a clear, transparent, and stable isotropic system. The desired method for formulating a nanoemulsion is a high-energy input in the system, but a significant literature also supports alternative methods, such as aqueous titration [21]. As reported, the low-energy methods (aqueous titration) are comparatively simple and can be used to produce fine droplet nanoemulsions [22].

### 2.6. Physical Stability Studies of the Developed Nanoemulsion

#### 2.6.1. Centrifugation

To determine metastable systems, the optimized nanoemulsion was centrifuged at 3500 rpm for 30 min, and those that did not show any change in the homogeneity were taken for the freeze–thaw stress test [23].

#### 2.6.2. Heating Cooling Cycle

Nanoemulsion formulations were subjected to six cycles between refrigerator temperatures of 4 and 45 °C (storage not less than 48 h at each temperature). Visual inspection was performed at different intervals for the stable formulations that were subjected to a centrifugation test. The nanoemulsion formulation was checked for temperature stability and was kept at three different temperature ranges of 2 to −8 °C (refrigerator), room temperature, and incubation at 40 °C and observed for any possible change, such as phase separation, flocculation, and precipitation [23].

#### 2.6.3. Freeze–Thaw Cycle 

The freeze–thaw cycle is one of the basic parameters to be carried out for a nanoemulsion study, where we need to take freshly prepared O/W nanoemulsion in a glass vial in a freezer for approximately 20 h and then transfer the same nanoemulsion to an oven at a fixed temperature of 40 °C, which is called thawing phase. It needs to be observed that if there is no phase separation, then it is sent back to the freezer (up to 10 cycles). Further, the formulations are observed for any change in phase separation, and other stability parameters need to be observed; for example, there should not be any conglomeration of particles that can be measured using a zetasizer [23,24].

### 2.7. Characterization of Minocycline-Loaded Nanoemulsion

#### 2.7.1. Globule Size and Polydispersity Index (PDI)

Globule size is a critical parameter that may influence the composition and bioacceptability of a nanoemulsion. The PDI mainly determines sample uniformity in terms of either monodisperse or polydisperse particle in a nanoemulsion. The determination of both was performed using Zetasizer Nano ZS90, Malvern Instruments, Malvern, UK) at 633 nm [25]. The different samples were analyzed in triplicate, and the results were described as mean ± standard deviation.

#### 2.7.2. Zeta Potential

The zeta potential of an optimized nanoemulsion was measured by a dynamic light scattering technique, Zetasizer Nano (Malvern Instruments, Ltd., Malvern, UK), at room temperature and applied electric field 1 V. Prior to analysis, the formulation sample was dispersed in a prefiltered double distilled water at a ratio of 1:100. The analysis was performed in triplicate, and the result was described as mean ± standard deviation [26].

#### 2.7.3. Refractive Index

The Abbe-type refractometer (Precision Standard Testing Equipment Corp., Berlin, Germany) determined the refractive index of the nanoemulsion formulation. The measure of the speed of light through a substance is called refractive index. The more is the time taken by light to travel through the media, the higher is the value [27].

#### 2.7.4. Drug Content

An amount of 1 mL of the prepared nanoemulsion formulation was taken and added into the methanol. Absorbance was determined using a UV spectrophotometer [28] at a lambda max of 254 nm. 

#### 2.7.5. Differential Scanning Colorimetry

A differential scanning calorimeter was used for thermal analysis, which suggests the mechanism associated with the destabilization of the nanoemulsion. The sample to be analyzed was crimped nonhermetically in an aluminum pan and heated from 30 to 200 °C. There was an increase in temperature, which was recorded as 10 °C/min, while the nitrogen flow rate was maintained at 60 mL/min in the instrument. An amount of 5% mannitol was used as a cryoprotectant for lyophilization [29].

#### 2.7.6. Transmission Electron Microscopy

To investigate the nanoemulsions’ morphological characteristics and to confirm the size measurement results, TEM (Zeiss, EM10C, 80 kV, Oberkochen, Germany) was employed. A sample preparation for TEM was performed by placing one drop of 100 times diluted sample on the copper grid. Samples were stained with uranyl acetate, and a dried copper grid was placed for TEM analysis [30].

### 2.8. Development of Nanoemulgel

A nanoemulgel containing minocycline for topical application was prepared by using Carbopol 940 as a gelling agent at different concentrations (0.5–1.0%). A minocycline nanoemulgel was prepared by dissolving 1.0% Carbopol 940 in purified water and left for complete swelling. TEA (triethanolamine (1–1.5% *v*/*v*)) was added in order to get the gel matrix. The transparent hydrogel was obtained after thoroughly mixing. Further in this gel, 20.0% *v*/*w* optimized minocycline nanoemulsion was incorporated to obtain a minocycline-loaded nanoemulgel preparation.

### 2.9. Evaluation of Formulated Nanoemulgel

#### 2.9.1. Measurement of pH

A digital pH meter was used to determine the pH of various gel formulations. One gram of gel was dissolved in 100 mL distilled water and stored for 2 h [31]. 

#### 2.9.2. Viscosity Study

A Brookfield viscometer is used for the determination of the viscosity of the pre-pared gel. The gels were rotated at 80 rotations per minute, carried out for 160 s. At each speed, the corresponding dial reading was noted. 

#### 2.9.3. Spreadability

It helps in the determination of the extent of the area to which the gel readily spreads after application to the skin or affected part. The higher the spreading value, the more efficacious the formulation will be. A spreadability test was carried out by using an apparatus suggested by [32]. It is a simple device that consists of a wooden block and a pulley attached to it at one end. An excess amount of the gel was placed between two slides. ‘Slip’ and ‘Drag’ are characteristics of a nanoemulgel, by which the spreadability measurement was performed. One glass slide was fixed, and another was attached to a pulley. On top of the two slides, a 500 g weight was applied to obtain the uniform film. The upper slide moved with the application of the weight to it through the pulley, and amount of the spread gel was noted.

#### 2.9.4. Texture Analysis

The textural properties, such as rupture strength, brittleness, and adhesiveness of a nanogel, were determined using the Texture Analyser TA.XT Plus (Stable Micro Systems, Godalming, UK). Five replicate analyses were performed at room temperature for each formulation, providing the same conditions, such as speed, rate, and depth of the insertion for each measurement. [33].

### 2.10. In Vitro Drug Release

The in vitro drug release profile of an optimized minocycline nanoemulsion (20 mL) and minocycline nanoemulgel (equivalent to 20 mL nanoemulsion) was carried out using a semipermeable dialysis membrane of average diameter. The dialysis membranes were immersed in a beaker containing 100 mL PBS (pH 5.5) on a magnetic stirrer (100 rpm) at 37 ± 0.2 °C for 24 h. An aliquot (3 mL) was withdrawn at predetermined time intervals and replaced with an equal volume of PBS to maintain a sink condition. The drug concentration in the samples withdrawn was analyzed spectrophotometrically at λmax 274 nm. The test was performed in duplicate [25].

### 2.11. Ex Vivo Permeation Study

The ex vivo skin permeation studies of a minocycline nanoemulgel and minocycline nanoemulsion were conducted using a Franz diffusion cell. Accurately weighed formulation samples were placed in a donor compartment, and the receptor compartment was filled with 10 mL of phosphate buffer saline of pH 5.5. A stratum corneum of 1 mm thickness was prior obtained from female Wistar rats weighing 200–250 g at 3–4 months. The skin was carefully excised, washed with saline, and placed between the compartments. The prepared assembly was placed on a magnetic stirrer, 50 rpm, and maintained at 37 ± 1 °C for 8 h [34].

### 2.12. Confocal Laser Scanning Microscopy (CLSM)

The excised rat skin samples were placed between the donor and receptor compartments of the Franz diffusion cell. The donor compartment was filled with a rhodamine nanoemulgel, and the receptor compartment was filled with PBS. The skin sample was removed after 24 h, washed with distilled water and alcohol to remove the excess stain, and evaluated using CLSM (Olympus FluoView^TM^ FV1000, Hamburg, Germany) at excitation and emission wavelengths of 540 and 625 nm, respectively [33].

## 3. Results and Discussion

### 3.1. Screening of Oils

The high capacity of oil to solubilize a drug plays a critical role as the more the solubilization more can be the target dose incorporated. For the present formulation, minocycline was taken as a drug of choice, which has low lipophilicity (log *p* = 0.36 ± 0.002) [35]. This attribute of a drug served as a primary criterion for the selection of oils as the oil capable of solubilizing even the poor lipophilic drug at low concentrations could be more suitable than the one either poorly solubilizing or solubilizing at higher concentrations. The solubility of minocycline was found to be highest in eucalyptus oil (4.6280 mg/mL) (Table 1).

### 3.2. Screening of Surfactants

Surfactants, as discussed earlier, may cause toxicity if used in larger concentrations than prescribed limits. For the present formulation, the major concern could be an irritation to an already-compromised skin. Thus, the surfactants having a safe skin profile were considered for the present work, and the selection was performed on the basis of drug solubilization capacity. The solubility of minocycline was found to be highest in Tween 20 (14.35563 mg/mL) (Table 2), which was selected as a surfactant [36].

### 3.3. Screening of Cosurfactants

Cosurfactants are also an integral part for nanoemulsion systems as they may affect the phase separation behavior. The solubility of minocycline was found to be highest in Transcutol HP (4.81338 mg/mL) (Table 3).

### 3.4. Pseudo-Ternary Phase Diagram

**Figure 1:**
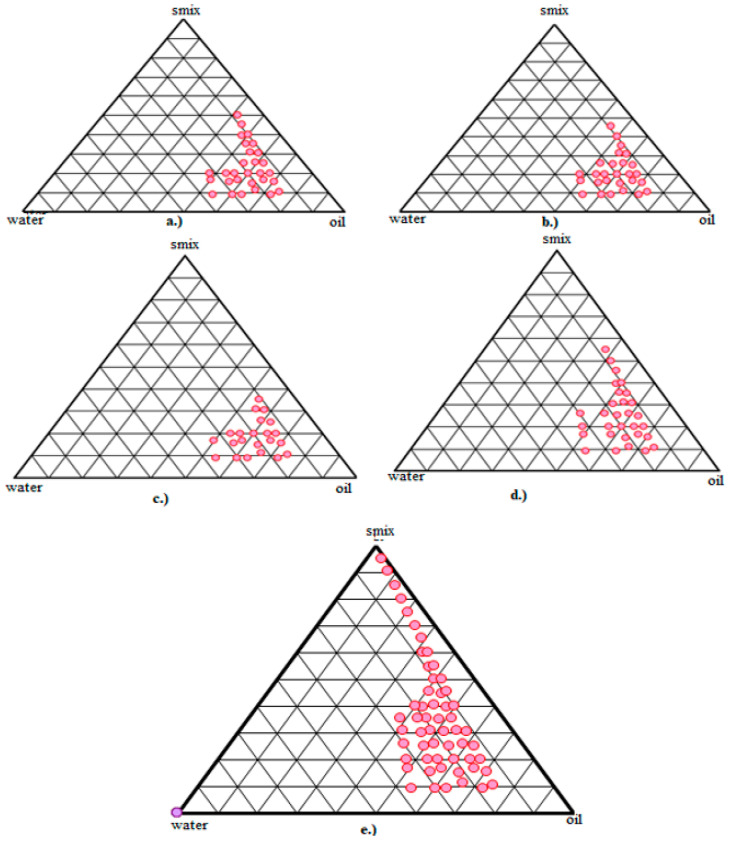
Pseudo-ternary phase diagrams showing different Smix ratios of the surfactant/cosurfactant ratio, (**a**) 1:1; (**b**) 1:2; (**c**) 2:1; (**d**) 4:1; (**e**) 5:1, of the nanoemulsion, which is composed of Tween 20 (surfactant), Transcutol HP (cosurfactant), eucalyptus oil as oil phase, and water (q.s).

Pseudo-ternary phase diagrams were constructed to study the existence of the nanoemulsion formation zone. It was constructed using eucalyptus oil, Tween 20, and Transcutol HP as the oil, surfactant, and cosurfactant, respectively. Minocycline with Transcutol HP was dropped in a vial containing a mixture of Tween 20 and oil. Water was added continuously to the mixture, and as the water was added, there were transitions visible from opaque to translucent and, finally, transparent [37]. For the optimization of the nanoemulsion, varied ratios of surfactant and cosurfactant (Smix) were used, and their effect on nanoemulsion formation was assessed. As shown in Figure 1, a Smix ratio of 5:1 was found in the phase diagram with a maximum o/w nanoemulsion region compared with the nanoemulsion made with other ratios of the Smix.

### 3.5. Dispersion Stability Study

The dispersion stability for the present formulation was performed qualitatively on a visual parameter of either phase separation or no phase separation in various stress tests, as presented in Table 4.

Following visual observation, the formulations F7, F8, and F9 were found to be stable and selected for further characterization.

### 3.6. Characterization of Nanoemulsion

The average globule size and PDI of all the prepared nanoemulsions fall under the range of 90–201 nm and 0.3–0.6, respectively (Table 5). Although all three formulations showed globule size in the nanorange, the formulation F9 has shown the lowest particle range, that is, 91.11 nm and PDI of 0.550. The formulation F9 was selected for further studies.

#### 3.6.1. Zeta Potential

**Figure 2:**
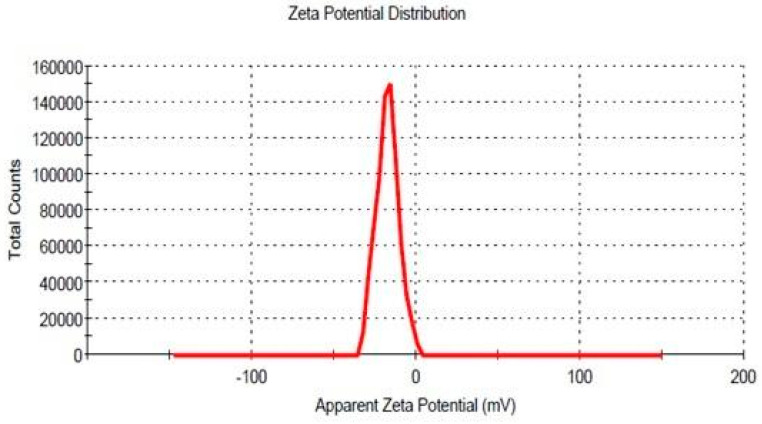
Zeta potential graph of optimized formulation.

The zeta potential of the formulation (F9) was found to be −17.40 mV (Figure 2), which falls under the range of +30–−30 mV. The presented range gives an idea for formulation stability in terms of less aggregation among the particles. 

#### 3.6.2. The Refractive Index

The refractive index of the selected nanoemulsion formulations (F9) was found to be 1.6.

#### 3.6.3. Drug Content

The drug content of the optimized nanoemulsion formulation (F9) was found to be 97.23 *w*/*v* ± 1.12%.

#### 3.6.4. Differential Scanning Calorimeter 

**Figure 3:**
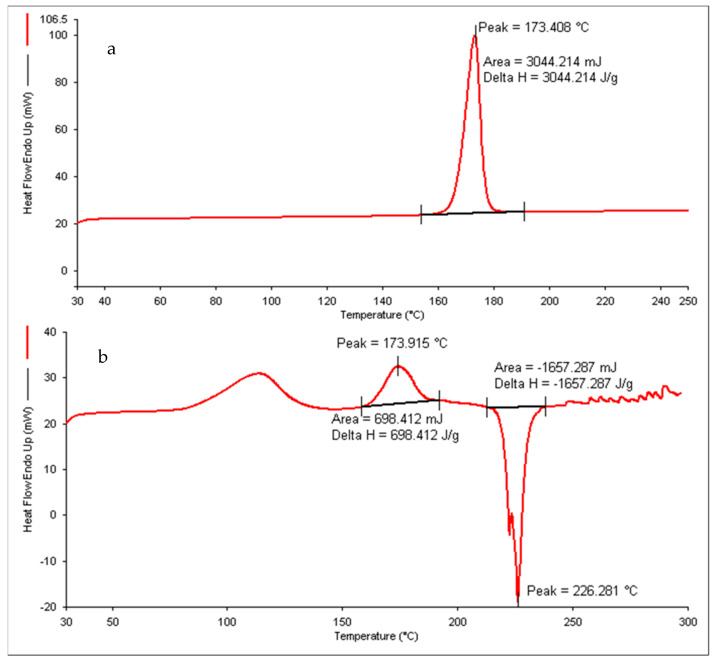
Differential scanning calorimetry (DSC) thermogram of (**a**) minocycline nanoemulgel (NEG) and (**b**) pure minocycline.

The DSC thermogram of pure drug minocycline exhibited the presence of an exothermic and endothermic peak at 163.5 and 219.1 °C, respectively, whereas the formulation (F-9) showed its peak at 173.408 °C, as shown in Figure 3. As no significant peak of drug minocycline was seen, this signifies that the drug is well encapsulated in the formulation [38].

#### 3.6.5. Transmission Electron Microscopy

**Figure 4:**
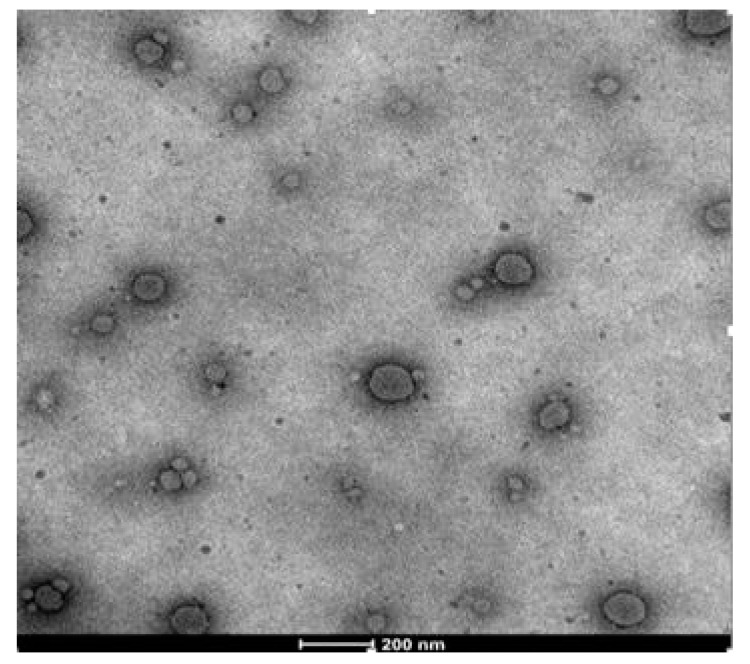
TEM image of nanoemulsion.

The TEM image of minocycline nanoemulgel particles in Figure 4 represents a spherical morphology with uniform size distribution. The particle size observed was in good agreement with the size obtained by a zetasizer (Nano-ZS90, Malvern Instruments, and Worcestershire, UK). The technique of Cryo TEM was used as it is available at the Sophisticated Analytical Instrument Facility (SAIF), All India Institute of Medical Sciences (AIIMS), New Delhi. The energy used in TEM was reported to be 40–120 kV. The effect of TEM on the structure of these soft materials is the visualization of their supramolecular organization with defined microscopic properties and macroscopic characteristics [39].

### 3.7. Characterization of Nanoemulgel and Development of Nanoemulgel

#### 3.7.1. Development of Nanoemulgel

A nanoemulgel was prepared using Carbopol 940 at a concentration of 1.0% *v*/*v* and triethanolamine at a concentration of 1% *w*/*w* to derive a clear gel. 

#### 3.7.2. Measurement of pH

The pH of the formulated gel was found to be 5.40, which is in the desirable range for skin care products (pH 4–7).

#### 3.7.3. Spreadability 

The spreadability of the gel is a critical parameter for better absorption of the drug. The formulated gel was found to be 5 cm/s spreadable by applying 500 gm of shear.

#### 3.7.4. Texture Analysis

Texture profile analysis was performed for the optimized gel to evaluate its consistency, firmness, and cohesiveness. The firmness of the sample was found to be 70.56 g ± 0.66. Consistency was found to be 112.20 g s ± 0.021, which indicates a high sample consistency, and cohesiveness was found to be −35.90 g ± 0.041, which suggests a high cohesive nature of the gel. The above-mentioned parameters have the following real-life meaning as these ingredients must provide adequate firmness so that the product is not too thick to cause difficulty in extrusion from the container, and important parameters of firmness and consistency were observed, which indicate that the force needed to deform the gel became higher, thus determining better spreadability and ease of application on the skin. Cohesiveness is the negative peak that was recorded during the movement of the probe upwards and is related to the intramolecular forces of the nanoemulgel [39].

### 3.8. In Vitro Drug Release

**Figure 5:**
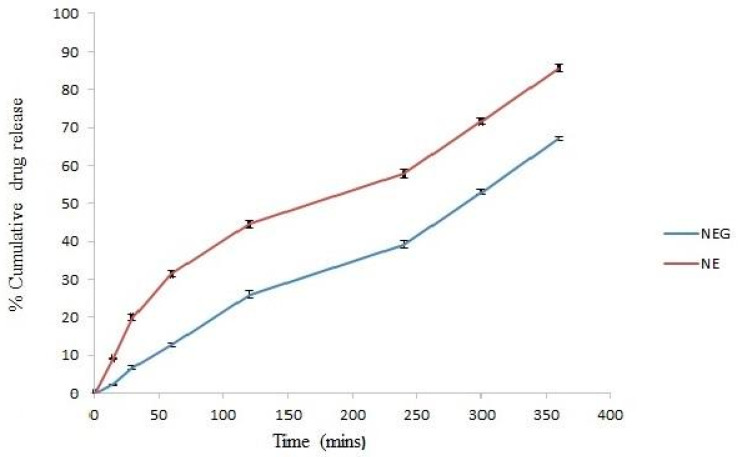
Release profile of minocycline nanoemulsion (NE) and minocycline nanoemulgel (NEG).

The in vitro release of minocycline from the selected nanoemulsion and nanoemulgel was assessed using the dialysis bag method. As demonstrated in Figure 5, the drug release from the selected optimized nanoemulsion formulae was comparatively higher than that from the nanoemulgel under the same set of experimental conditions. It was observed that the optimized minocycline nanoemulsion releases the drug at a faster rate as compared with the nanoemulgel preparation. Therefore, the desirable sustained release behavior was exhibited by the minocycline nanoemulgel.

### 3.9. Ex Vivo Permeation Study

**Figure 6:**
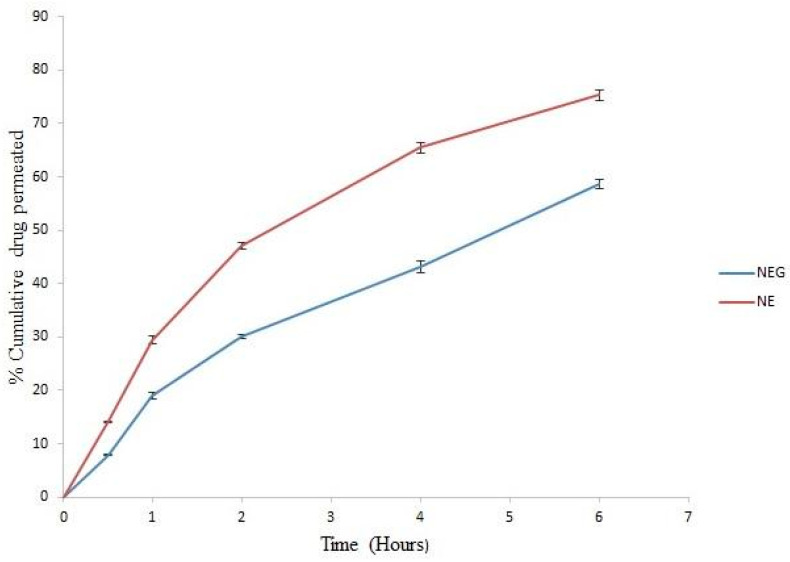
Permeation profile of minocycline nanoemulsion (NE) and minocycline nanoemulgel (NEG).

Ex vivo permeation studies were performed with the minocycline nanoemulsion and its gel formulation (strength equivalent to minocycline 1 mg per mL). The cumulative amounts of the drug permeated after 6 h from the minocycline nanoemulsion and minocycline nanoemulgel were 75% and 58%, respectively [29], as shown in Figure 6. This indicates that the nanoemulgel shows sustained release behavior and will give a better result after a long hour of usage on the skin.

### 3.10. Confocal Laser Scanning Microscopy

**Figure 7:**
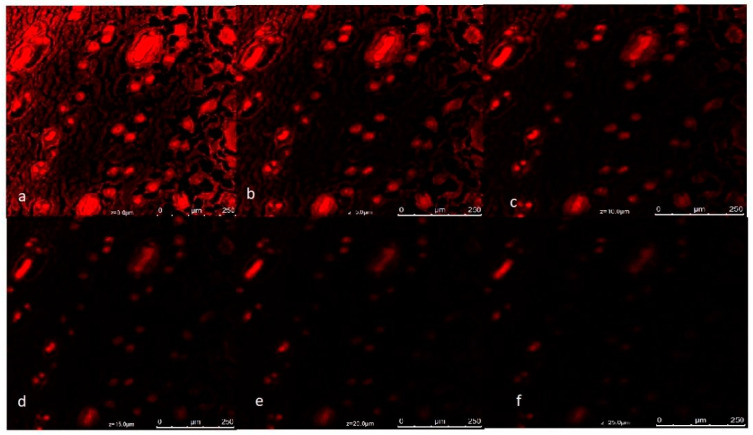
Confocal laser scanning microscopy (CLSM) images showing the penetration and distribution of the formulation at (**a**) 0, (**b**) 5, (**c**) 10, (**d**) 15, (**e**) 20, and (**f**) 25 µm.

CLSM reveals fluorescence images of the dorsal skin of a rat after being mounted on the Franz diffusion cell for a permeation study with rhodamine-loaded NEG. After 24 h, the fluorescence images were taken. The amount of the drug released from the nanoemulgel mainly depends on the organic content in it. The higher is the amount of oil, the greater is the drug release. As shown in Figure 7, dye penetration was observed up to 25 µm, which is expected to be sufficient for its therapeutic activity [40].

## 4. Conclusions

Acne rosacea is considered a general category disease, but if left untreated, it leads to worst cases, particularly in males, termed rhinophyma. The above collective results suggest that the prepared o/w nanoemulgel of minocycline can provide sustained release topically. The various characterization parameter of the formulation indicates that the nanoemulgel was successfully developed and optimized. Drug release and permeation profile suggested that the formulation is capable enough for the treatment being a better modality and locoregional targeted drug delivery system. Henceforth, the proposed minocycline-bearing nanoemulgel is expected to treat acne rosacea with better therapeutic outcomes.

## Figures and Tables

**Table 1:** Solubility of minocycline in different oils.

S. No.	Oils	Drug Solubility (mg/mL) ± SD (N = 3)	Density	Molecular Formula
1.	Olive oil	0.3145 mg/mL ± 0.003	0.920 g/mL	C_88_H_164_O_10_
2	Rose oil	0.58802 mg/mL ± 0.23	0.964 g/mL	NA
3.	Castor oil	1.71409 mg/mL ± 0.46	0.955 g/mL	C_57_H_104_O_9_
4.	Eucalyptus oil	4.6280 mg/mL ± 0.78	0.909 g/mL	C_10_H_18_O

**Table 2:** Drug solubility in surfactant.

S. No.	Surfactants	Drug Solubility (mg/mL) ± SD (N = 3)	Density	Molecular Formula
1.	Tween 20	14.35563 mg/mL ± 0.78	1.11 g/mL	C_26_H_50_O_10_
2.	Cremophor EL	9.81205 mg/mL ± 0.06	1.05 g/mL	C_5_H_12_O_4_
3.	Tween 40	6.82042 mg/mL ± 0.74	1.083 g/mL	C_12_H_18_O_11_
4.	Tween 80	6.04502 mg/mL ± 0.88	1.08 g/mL	C_24_H_44_O_6_

**Table 3:** Drug solubility in co-surfactant.

S. No.	Cosurfactants	Drug Solubility (mg/mL) ± SD (N = 3)	Density	Molecular Formula
1.	Transcutol HP	4.81338 mg/mL ± 0.87	NA	NA
2.	Ethanol	3.8204 mg/mL ± 0.54	0.789 g/mL	C_2_H_6_O
3.	Propylene glycol	3.81205 mg/mL ± 0.06	1.036 g/mL	C_3_H_8_O_2_
5.	Propan-2-ol	2.04502 mg/mL ± 0.67	0.785 g/mL	C_3_H_8_O

**Table 4:** Dispersion Stability of formulations.

Smix	Batch No.	Centrifugation	Heating Cooling	Freeze–Thaw Cycle
1:2	1:3 (F1)	No phase separation	No phase separation	Hazy solution
	1:4 (F2)	No phase separation	Complete phase separation	No phase separation
	1:5 (F3)	Hazy solution	No phase separation	Hazy solution
3:1	1:3 (F4)	Hazy solution	Hazy solution	Hazy solution
	1:4 (F5)	Hazy solution	No phase separation	Complete phase separation
	1:5 (F6)	No phase separation	No phase separation	No phase separation
5:1	1:3 (F7)	No phase separation	No phase separation	No phase separation
	1:4 (F8)	Hazy solution	No phase separation	Hazy solution
	1:5 (F9)	No phase separation	No phase separation	No phase separation

**Table 5:** Comparative analysis of the globule size and PDI for different formulations.

Formulation	Globule Size	PDI
	Mean ± SD	Mean ± SD
	(N = 3) (nm)	(N = 3)
F6 (3:1) Smix Ratio (1:5)	200.2 ± 0.899	0.348 ± 0.003
F7 (5:1) Smix Ratio (1:3)	175.0 ± 0.205	0.563 ± 0.004
F9 (5:1) Smix Ratio (1:5)	91.11 ± 0.028	0.550 ± 0.002

## Data Availability

The data presented in this study are available in this article.
